# A Learned-SVD Approach to the Electromagnetic Inverse Source Problem

**DOI:** 10.3390/s24144496

**Published:** 2024-07-11

**Authors:** Amedeo Capozzoli, Ilaria Catapano, Eliana Cinotti, Claudio Curcio, Giuseppe Esposito, Gianluca Gennarelli, Angelo Liseno, Giovanni Ludeno, Francesco Soldovieri

**Affiliations:** 1 Dipartimento di Ingegneria Elettrica e delle Tecnologie dell’Informazione (DIETI), Università di Napoli Federico II, Via Claudio 21, I 80125 Napoli, Italy; el.cinotti@studenti.unina.it (E.C.); clcurcio@unina.it (C.C.); angelo.liseno@unina.it (A.L.); 2 Consiglio Nazionale delle Ricerche, Istituto per il Rilevamento Elettromagnetico dell’Ambiente (IREA), Via Diocleziano 328, I 80124 Napoli, Italy; catapano.i@irea.cnr.it (I.C.); esposito.g@irea.cnr.it (G.E.); gennarelli.g@irea.cnr.it (G.G.); ludeno.g@irea.cnr.it (G.L.); soldovieri.f@irea.cnr.it (F.S.)

**Keywords:** autoencoder, deep neural networks, inverse source, singular value decomposition, learned singular value decomposition

## Abstract

We propose an artificial intelligence approach based on deep neural networks to tackle a canonical 2D scalar inverse source problem. The learned singular value decomposition (L-SVD) based on hybrid autoencoding is considered. We compare the reconstruction performance of L-SVD to the Truncated SVD (TSVD) regularized inversion, which is a canonical regularization scheme, to solve an ill-posed linear inverse problem. Numerical tests referring to far-field acquisitions show that L-SVD provides, with proper training on a well-organized dataset, superior performance in terms of reconstruction errors as compared to TSVD, allowing for the retrieval of faster spatial variations of the source. Indeed, L-SVD accommodates a priori information on the set of relevant unknown current distributions. Different from TSVD, which performs linear processing on a linear problem, L-SVD operates non-linearly on the data. A numerical analysis also underlines how the performance of the L-SVD degrades when the unknown source does not match the training dataset.

## 1. Introduction

An electromagnetic inverse source problem [1] consists of determining an electric/magnetic current source from the radiated field over a given measurement domain.

From a mathematical viewpoint, the inverse source problem is stated as the inversion of the linear operator mapping the source (current) space onto the data (radiated field) one. As is well–known, this problem is ill-posed [2,3,4], and regularization is necessary to achieve a physically meaningful solution. Classical regularization schemes include the Tikhonov method [5], Truncated Singular Value Decomposition (TSVD) [6], Total Variation (TV) [7], the Lasso scheme [8], iterative methods [9], etc. Among these, TSVD is usually exploited when the singular values of the radiation operator exhibit a step-like behavior [6].

Recently, Deep Learning (DL), initially developed for image processing and computer vision, has become much more affordable and increasingly popular for solving inverse imaging problems (e.g., see [10,11,12,13,14,15,16,17,18]). Unlike canonical methods, which exploit the explicit mathematical expression of a linear operator between subspaces, Deep Neural Networks (DNNs) instead leverage large datasets to learn the solution to the inverse problem. As an example, Convolutional Neural Networks (CNNs) have reported improvements as compared to state-of-the-art methods in various tasks such as denoising, deconvolution, super-resolution, and medical imaging [10]. Concerning electromagnetic inverse problems, most of the attention has been focused on inverse scattering [13,15,16,17,19]. Interesting review articles on this topic have also been recently published [11,18,20]. Within the antennas and propagation community [21], the potentialities of Artificial Intelligence have been explored for the optimization of radio propagation in communication channels [22,23] and in the framework of antenna synthesis [24] and diagnostics [25], just to mention a few.

In [13,15,16,17], CNNs were adopted to solve a 2D electromagnetic inverse scattering problem, exploiting different topologies and learning schemes. In [26], for the very first time, a DL technique was applied to the solution of a 2D electromagnetic inverse source problem using U-Net. Following this work, in [27], the Learned Singular Value Decomposition (L-SVD) approach, recently introduced in [28] and later applied to diffuse optical tomography [29], was applied to the same problem for the first time.

Here, we investigate the performance of L-SVD for a 2D electromagnetic inverse source problem and compare it against the classical TSVD regularization scheme. In detail, we consider the radiation operator, say A, that is based on an integral relationship linking the unknown current to the field data. As long as A acts between linear subspaces, as in the case of the TSVD, the modelling of the physical phenomenon is linear. By contrast, DNNs are trained to operate on input–output sets of A, defined by a discrete number of test cases, which are not subspaces. Therefore, restricting the inputs and outputs of A to sets instead of subspaces breaks down the linearity of the inverse model. From this point of view, the interest in understanding the performance of L-SVD for an inverse source (dealt with as being non-linear) stems from the fact that such a supervised learning technique can be considered as an alternative to the classical SVD to include some kinds of non-linearity in the inverse modelling. It should be also noticed that, assuming the unknown belongs to a subspace is in principle unrealistic, since real sources and fields have limited energy. For example, arbitrarily large currents, which are admissible in the subspace assumption, should be ruled out in practical applications. It should be also observed that letting the unknown to be arbitrarily large opens the space to ill-positioning/ill-conditioning. Minimum norm solutions or similar strategies (e.g., Tikhonov regularization) operate with the purpose of dismissing arbitrarily large unknowns, thus preventing the noise contribution from blowing up during the inversion.

The L-SVD architecture consists of three interconnected DNNs: a data autoencoder (AE), a source AE, and a scaling layer establishing the connection between the data and the source latent spaces. The three DNNs perform non-linear processing on the input data according to the underlying non-linear point of view of the inverse source problem. Numerical simulations demonstrate that, with proper training on a well-organized dataset, L-SVD outperforms classical TSVD by allowing significantly lower reconstruction errors, as long as the unknown is accounted for by the training dataset. Furthermore, L-SVD retrieves faster spatial variations of the source, with a consequent enhancement in the spatial resolution as compared to TSVD. This is due to intrinsic non-linearity implemented in the activation functions of the L-SVD. In other words, while TSVD applies linear processing on the data, the L-SVD applies a non-linear one. The numerical analysis also underlines how the performance of L-SVD degrades when the unknown source does not match the dataset used for the training.

It should be pointed out that a DL approach was recently presented, exploiting the use of AEs [30]. The input of the DNN is the pre-processing result provided by the TSVD reconstruction worked out from the available radiation data, and the DNN is appointed to improve the quality (e.g., resolution) of such a reconstruction [30]. By contrast, L-SVD directly adopts the available radiated field as the input and does not need the TSVD pre-processing. We also mention that the capability of AEs to separate the background and anomalies for complex scenes was recently exploited for anomaly detection in hyperspectral images [31], improving the performance of low-rank and sparse matrix decompositions [32], also with the aid of priors arising from the use of the Robust Principal Component Analysis [33].

We note that the electromagnetic inverse source problem studied in this paper is not merely theoretical but has implications in various practical applications. For example, antenna analyses [34] and characterization [35] and diagnostics [36] require the determination of a radiating current from near-field data; the localization of radiating sources in radio frequency localization problems [37] or of scattering sources in through-the-wall imaging [38] require the determination of the support of sources or equivalent sources, respectively; formulating the Ground-Penetrating Radar (GPR) imaging problem as an inverse source one can help interpret the acquired data [39]; finally, the inverse source problem can help in understanding some features of inverse scattering [1]. We also note that the use of DNN in electromagnetic inverse problems is not yet widespread throughout the literature, much less for electromagnetic inverse source problems, and so DNNs for electromagnetic sources are a timely problem to be investigated.

The paper is organized as follows. Section 2 formulates the inverse source problem and recalls the SVD approach for linear problems. In Section 3, the L-SVD architecture is detailed. Numerical simulations are carried out in Section 4. In Section 5, we discuss the results and point out the potentialities and limitations of L-SVD. Finally, conclusions follow in Section 6.

## 2. Inverse-Source Formulation for TSVD

As already mentioned in the Introduction, we face an inverse source problem using a DNN; as a result, in this Section, we present the formulation thereof. To avoid irrelevant technicalities, a 2D problem is addressed, with data collected in the far-field zone, so as to reach a Fourier transform relation between unknown and data. Such a mapping is particularly useful to study, thanks to the availability of analytical results and benchmarks, and is amenable to immediate extensions for other applications, as mentioned in Section 5. Furthermore, the 2D problem is considered for a planar source which can model either the case of primary sources or secondary sources arising from the application of the equivalence theorem [40].

**Figure 1:**
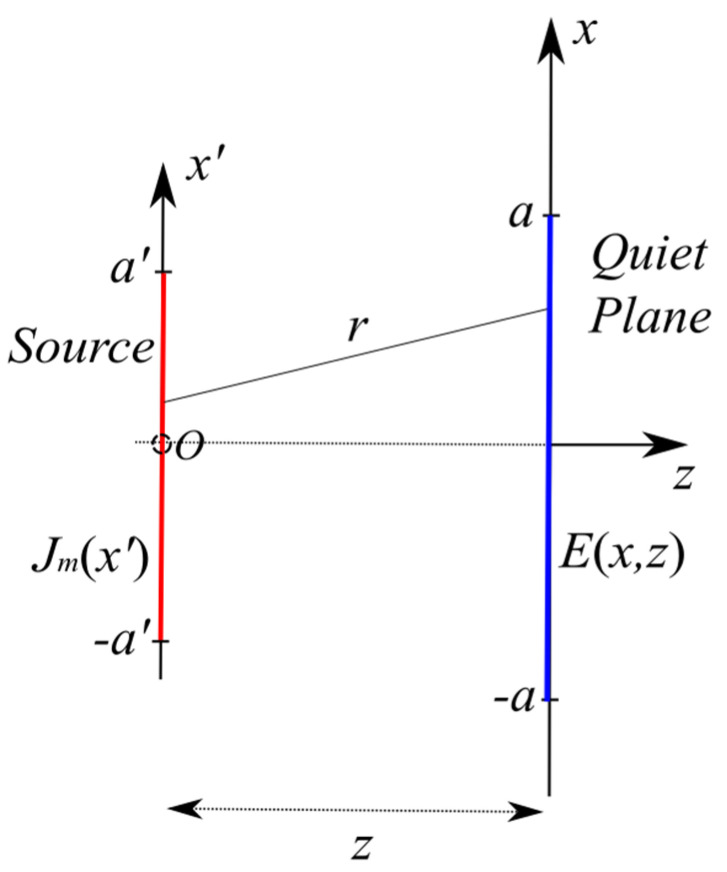
Geometry of the inverse source problem.

Therefore, let us consider the 2D scalar problem represented in Figure 1, where a rectilinear magnetic current source $J_{m}$ radiates in free space. The current is directed along the *x*-axis, namely, $J_{m}=J_{m}{\hat{i}}_{x}$, and has support $\left[-a^{\prime },\;a^{\prime }\right]$. The observation domain is also rectilinear with extent 2a along the *x*-axis, set at distance z apart from the source, and is centered with respect to it. The $e^{j\omega t}$ time dependence is assumed and dropped out.

According to [6], the relationship between the *y* component of the radiated electric field E and the magnetic current source $J_{m}$ writes as

(1) $$E\left(x,z\right)=\int_{-a}^{a}G\left(x-x^{\prime },z\right)J_{m}\left(x^{\prime }\right)dx^{\prime }=AJ_{m}\;$$

where G is the magnetic–electric Green’s function

(2) $$G\left(x-x^{\prime },z\right)=j\beta /4\frac{{H_{1}}^{\left(2\right)}\left(\beta r\right)z}{r},$$

${H_{1}}^{\left(2\right)}\left(\cdot \right)$ is the Hankel function of the second kind and first order, β=2π/λ is the free-space propagation constant, λ is the wavelength, and $r=\sqrt{{\left(x-x^{\prime }\right)}^{2}+z^{2}}$ is the distance between the observation point (x,z) and a generic source point (x′, 0). Equation (1) defines the unknown-to-data link and can be seen as a linear transformation, A:X→Y, mapping the space of the magnetic current sources X onto the data space Y. Both X and Y are assumed to be ${ℒ}^{2}$ spaces, i.e., spaces of square integrable functions. As long as E and $J_{m}$ are assumed to belong to a subspace, their relative mapping is linear.

The radiation operator A is compact, and, accordingly, it can be described by resorting to the SVD approach [4,6]. Specifically, we denote, with ${\left\{\sigma_{p};u_{p};v_{p}\right\}}_{p=0}^{\infty }$, the singular spectrum of A, where $\sigma_{p}$ are the singular values, and $u_{p}$ and $v_{p}$ form orthonormal basis functions in the spaces of data and unknowns, respectively. Due to the ill-posedness of the problem, the singular values of A exhibit an exponential decay [6]. Therefore, an approximate regularized solution to the inverse source problem can be obtained by resorting to the TSVD inversion scheme [3]. For the cases of our interest, the singular values exhibit a step-like behavior, so that a regularized inversion performed with a Tikhonov-like weighting of the singular values shows performance similar to that achieved by the TSVD approach [4].

In the case of an observation domain located in the far-field zone and paraxial approximation, Equation (1) rewrites as follows [6]

(3) $$E\left(x,z\right)={\left[\frac{j}{4\lambda z}\right]}^{\frac{1}{2}}e^{-j\beta \rho_{0}}\int_{-a\prime }^{a\prime }J_{m}\left(x^{\prime }\right)\;e^{j\left[\frac{2\pi xx^{\prime }}{\lambda z}\right]}dx^{\prime }=AJ_{m}\;$$

with $\rho_{0}≃\sqrt{x^{2}+z^{2}}$.

Based on Equation (3), the radiation operator A is now in the form of a Fourier transform. As a result, its singular spectrum can be expressed in closed form as follows [6,41,42,43]:

(4) $$u_{p}\left(x^{\prime }\right)=\frac{1}{\sqrt{\chi_{p}\left(c^{\prime }\right)}}\psi_{p}\left(c^{\prime };x^{\prime }\right)\;\;$$

with

(5) $$\sigma_{p}=\sqrt{\chi_{p}\left(c^{\prime }\right)/4}\;\;$$

and

(6) $$v_{p}\left(x\right)=\frac{e^{-j\beta \rho_{0}}j^{p+\frac{1}{2}}\sqrt{\frac{a}{a^{\prime }}}\;\psi_{p}\left(c^{\prime };\;x\frac{a}{a^{\prime }}\right)}{\sqrt{\chi_{p}\left(c^{\prime }\right)}}\;\;$$

where $\psi_{p}\left(c^{\prime };x^{\prime }\right)$ and $\chi_{p}\left(c^{\prime }\right)\;\left(n=0,\;1,\;\ldots \right)$ are the p-th prolate spheroidal functions [41,42] and their corresponding eigenvalues; and $c^{\prime }=\frac{4aa^{\prime }}{\lambda z}$ is the so-called space-bandwidth product. Several properties of the prolate spheroidal functions are studied in [41,42]. However, they do not have closed-form expressions, and their determination requires the set-up of appropriate numerical algorithms. As mentioned, the eigenvalues $\chi_{p}\left(c^{\prime }\right)$ exhibit a “step-like” behavior, i.e., they are nearly constant up to a critical index, after which they exhibit an exponential decay. This index is interpreted as the (finite) Number of Degrees of Freedom (NDF) of the radiated field:

(7) $$NDF≃\frac{4aa^{\prime }}{\lambda z}\;\;$$

which is used as a truncation index $\bar{P}$ of the TSVD reconstruction formula.

A numerical evaluation of the SVD that is useful in the L-SVD context profits from a discretized counterpart of the integral Equation (3) with the method of moments [44] by adopting rectangular basis functions for the source domain and delta testing functions for the radiated field domain. To account for the presence of noise in the data, the following modelling is considered:

(8) $$E=\underline{\underline{A}}\;J_{m}+\eta$$

where $E\in {\mathbb{C}}^{M}$ and $J_{m}\in {\mathbb{C}}^{N}$ are the discretized data and source vectors; $\eta \in {\mathbb{C}}^{N}$ is the additive noise vector, assumed to be white Gaussian (AWGN); and $\underline{\underline{A}}\in {\mathbb{C}}^{M\times N}$ is the operator matrix. Here, $\underline{\underline{A}}$ is expressed in terms of its SVD as

(9) $$\underline{\underline{A}}=\underline{\underline{U}}\;\underline{\underline{S}}\;{\underline{\underline{V}}}^{H}$$

where H is the Hermitian conjugate, and $\underline{\underline{U}}\in {\mathbb{C}}^{M\times M}$ and $\underline{\underline{V}}\in {\mathbb{C}}^{N\times N}$ are the complex unitary matrices, whose columns are the left and right singular vectors $u_{p}$ and $v_{p}$, respectively. Moreover, $\underline{\underline{S}}\in {\mathbb{R}}^{M\times N}$ is a diagonal matrix, whose entries are the singular values $\sigma_{p}$, sorted in a decreasing order.

Since the matrix $\underline{\underline{A}}$ is ill-conditioned, the regularized source vector $\;{\hat{J}}_{m}$ achieved via TSVD is expressed as follows:

(10) $${\hat{J}}_{m}={\underline{\underline{V}}}_{r}\;{\underline{\underline{S}}}_{r}^{-1}{\underline{\underline{U}}}_{r}^{H}E=\overset{\bar{P}}{\underset{p=1}{\sum }}\frac{E^{T}u_{p}^{*}}{\sigma_{p}}v_{p}$$

where T denotes transposition; ${\underline{\underline{V}}}_{r}\in {\mathbb{C}}^{N\times \bar{P}}$; ${\underline{\underline{S}}}_{r}\in {\mathbb{R}}^{\bar{P}\times \bar{P}}$; ${\underline{\underline{U}}}_{r}\in {\mathbb{C}}^{M\times \bar{P}}$; and * is the conjugation operation.

## 3. L-SVD Reconstruction Approach

### 3.1. Mathematical Formulation

The L-SVD is a data-driven strategy based on a particular class of NNs, i.e., AEs. An AE learns to represent the input data in a lower-dimensional space (encoding), and then it reconstructs the original data from the encoded representation (decoding) [45]. The idea of encoding and decoding is somehow present also in the SVD when the data and the unknown are projected over the singular functions corresponding to the most significant singular values. The finite number of expansion coefficients represent the coding while using them to express the filtered version of the data, and unknowns represent the decoding process. The L-SVD paradigm was recently introduced in [28] to tackle inverse problems, where, as mentioned in Section I, the relationship between the data and the unknown is non-linear.

**Figure 2:**
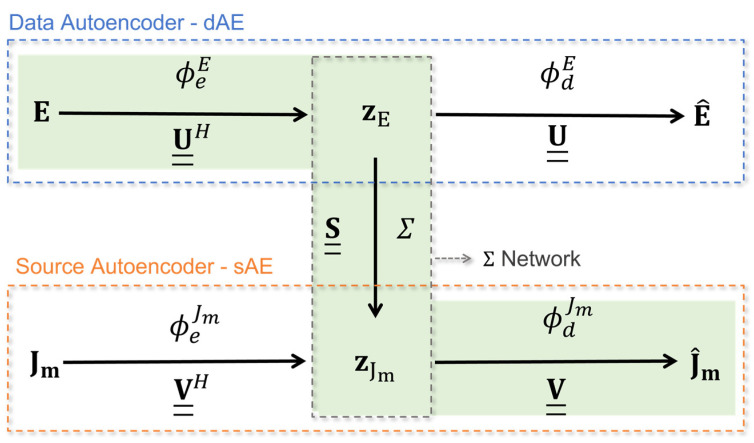
The L-SVD reconstruction approach and its parallelism with SVD.

As shown in Figure 2, the L-SVD strategy consists of three building blocks: (i) an AE operating on the data (dAE), (ii) an AE operating on the source (sAE), and (iii) a bridge network Σ creating a connection between the compressed source and data spaces. It is implicitly assumed that both AEs and Σ networks have a built-in non-linearity.

From a mathematical perspective, dAE can be defined through an encoder $\phi_{e}^{E}:Y\to Z^{E}$ mapping the data space Y onto a lower dimensional (latent) data space $Z^{E}\subseteq {\mathbb{R}}^{\bar{m}}$, $\bar{m}\leq M$ and by a decoder $\phi_{d}^{E}:Z^{E}\to Y$, which performs the inverse transformation. Therefore, the input data vector E can be reconstructed from its encoded feature (latent representation) $z_{E}\in Z^{E}$, i.e.,

(11) $$z_{E}=\phi_{e}^{E}\left(E\right),\;\hat{E}=\phi_{d}^{E}\left(z_{E}\right)$$

It must be noticed that the dAE plays the role of a denoising AE, when it is trained to provide a noise-free reconstruction $\hat{E}$ from a noisy input data vector E. However, the AE coding is lossy from the information theory point of view, and accordingly, a small but non-negligible reconstruction error arises even in the absence of noise in the data [45,46].

Similarly, sAE is defined by an encoder $\phi_{e}^{J_{m}}:X\to Z^{J_{m}}$ mapping the source space X onto a lower dimensional (latent) source space $Z^{J_{m}}\subseteq {\mathbb{R}}^{\bar{n}}$, $\bar{n}\leq N$, and by a decoder $\phi_{d}^{J_{m}}:Z^{J_{m}}\to X$, which performs the inverse transformation. Therefore, the source vector $J_{m}$ can be reconstructed from its latent code $z_{J_{m}}\in Z^{J_{m}}$, i.e.,

(12) $$z_{J_{m}}=\phi_{e}^{J_{m}}\left(J_{m}\right),\;{\hat{J}}_{m}=\phi_{d}^{J_{m}}\left(z_{J_{m}}\right)$$

Figure 2 highlights the parallelism between the L-SVD paradigm and the classical SVD, i.e., $\phi_{e}^{E}\;$ and $\phi_{d}^{E}$ play the role of the matrices ${\underline{\underline{U}}}^{H}$ and $\underline{\underline{U}}$, respectively. By analogy, with SVD, $\phi_{e}^{J_{m}}$ and $\phi_{d}^{J_{m}}$ play the role of the matrices ${\underline{\underline{V}}}^{H}$ and $\underline{\underline{V}}$, respectively.

The two latent codes $z_{E}$ and $z_{J_{m}}$ are related by a bridge operator $\Sigma :Z^{E}\mapsto Z^{J_{m}}$, such that $z_{J_{m}}=\Sigma \left(z_{E}\right)$, which plays the scaling role of the singular values in $\underline{\underline{S}}$ in the SVD approach.

The L-SVD reconstruction procedure is highlighted by the green path in Figure 2 and is summarized as follows:

1. Encoding the data E via the encoder $\phi_{e}^{E}$ to produce the latent code $z_{E}$ analogously to the product ${\underline{\underline{U}}}^{H}E$ of the SVD approach;
2. Connecting the latent codes $z_{E}$ and $z_{J_{m}}$ through the Σ operator, which mimics the SVD computation of ${\underline{\underline{S}}}^{-1}{\underline{\underline{U}}}^{H}E$;
3. Decoding the latent code $z_{J_{m}}$ with the decoder $\phi_{d}^{J_{m}},$ which corresponds to the final left multiplication by $\underline{\underline{V}}$ in the SVD.

**Figure 3:**
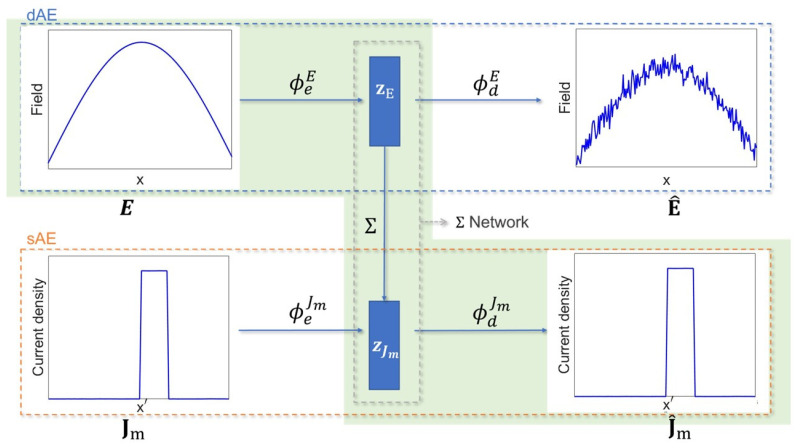
Representation of the L-SVD reconstruction strategy. The upper horizontal path refers to dAE, which takes noisy data in the input and provides denoised data as the output. The lower horizontal path represents the sAE, which reconstructs the ground truth from the originating source. The green path refers to the reconstruction path via the Σ network connecting the data and source latent spaces.

An illustrative example showing the operation of the L-SVD strategy is depicted in Figure 3, which was drawn from one of the cases considered in the numerical analysis, where the reconstruction process is highlighted with the green shaded area.

### 3.2. A Test Case and Dataset Generation

An L-SVD network was derived for a test case with the geometrical parameters detailed in Table 1.

Concerning the generation of the datasets necessary to train, validate, and test the L-SVD strategy, we assume the a priori information wherein the set of unknown magnetic current sources $J_{m}$ are modelled as rectangular pulses. Therefore, we set up the training dataset as a set of rectangular pulses having random positions $x_{0}$, widths *w*, and amplitudes *A*. Specifically, $x_{0}$ is treated as a uniform random variable within the interval [−5, 5] λ, *w* is a uniform random variable in [1, 5] λ, and *A* is a uniform random variable in the range [0.5, 1].

Once the dataset is generated, the corresponding noisy radiated field measurement is produced through Equation (8) with an additive white Gaussian noise (AWGN) characterized by a signal-to-noise ratio (SNR) equal to 30 dB. The dataset is partitioned as follows: 72,000 samples are used for training, 8000 are used for validation, and 20,000 samples are used for testing.

### 3.3. The TSVD Approach for the Considered Test Case

**Figure 4:**
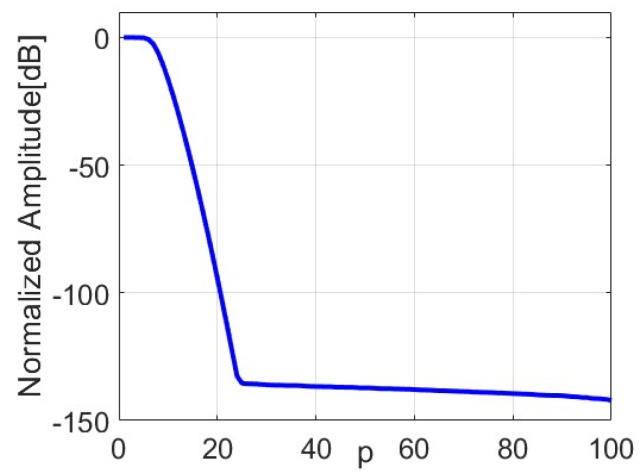
Normalized singular values (dB) of the operator matrix $\underline{\underline{A}}$.

Figure 4 shows the curve of the normalized singular values of the operator matrix $\underline{\underline{A}}$ obtained for the considered problem parameters. As expected, the singular values exhibit a step-like behavior, i.e., they are almost constant up to NDF = $4aa^{\prime }/\left(\lambda z\right)=6$, after which they show a rapid decay. This value is used as a truncation index in the TSVD inversion formula (10). It is further observed in Figure 4 that the exponential decay after the knee at p = 6 is not very fast, such that the first singular values after NDF (i.e., p≤10) are larger than −20 dB. The corresponding singular functions should then be incorporated in the regularization process, since they would provide a robust reconstruction against the noise. Note that the non-fast decay of the singular values is related to the non-large space–bandwidth product (see also [47]). The L-curve method [48] is also applied to verify the possibility of slightly refining the truncation index beyond NDF = 6. The L-curve is a log–log plot of the norm of the TSVD regularized solution ${\left\|{\hat{J}}_{m}\right\|}^{2}$ versus the norm of the residue $\|E-\underline{\underline{A}}\;{\hat{J}}_{m}{\|}^{2}$. The optimal truncation index $\bar{P}$ is the one achieved at the corner of the L-curve [45] and depends on the specific test case. More details will be provided in the Numerical Results Section.

### 3.4. Network Traning and Architecture

The L-SVD architecture is implemented through Multilayer Perceptrons (MLPs), which are fully connected feedforward NNs [49]. Since these networks are conceived to operate with real data, both data and source vectors are rearranged into real vectors by concatenating their real and imaginary parts. Therefore, we introduce the data and source vectors $E^{\prime },$ $\hat{E}\prime \in {\mathbb{R}}^{2M}$, $J_{m}^{\prime },$ and ${\hat{J}}_{m}^{\prime }\in {\mathbb{R}}^{2N}$, where the prime symbol indicates that the vectors are real-valued. The NNs’ topologies were determined by means of a parametric analysis involving different numbers of layers and nodes within each layer and different types of activation functions.

**Figure 5:**
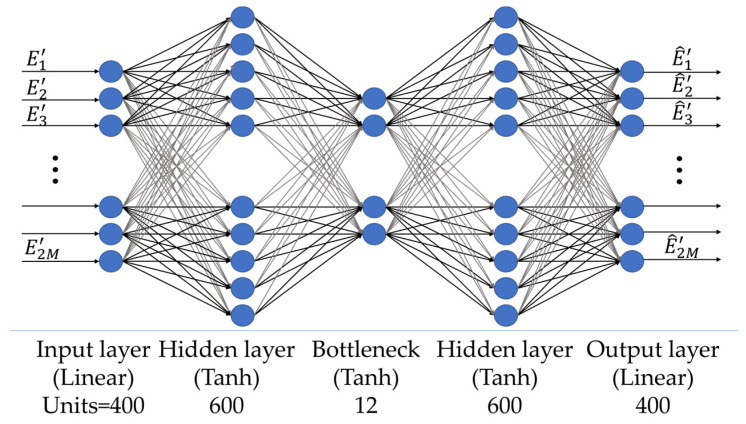
MLP architecture of the dAE.

As shown in Figure 5, $E^{\prime }$ and $\hat{E}\prime$ are the dAE input and output, respectively. The dAE is composed of 5 layers: 1 input layer, 3 hidden layers, and 1 output layer. The input layer has a number of nodes equal to 2M. Based on the discretization of the problem, we have 2M = 400 for the neural network set up in this work. The second layer has 600 nodes, and a hyperbolic tangent (Tanh) activation function [50] is applied at the output of each node. The third layer has a number of nodes equal to $b_{1}$ = 12 (bottleneck), which is twice the *NDF* value. A Tanh activation function is applied at the output of this layer, thus obtaining the latent representation of the data. The next layer consists of 600 nodes equipped with a Tanh activation function. Finally, the output layer has a number of nodes equal to the input (2M = 400), and a linear activation function is used.

**Figure 6:**
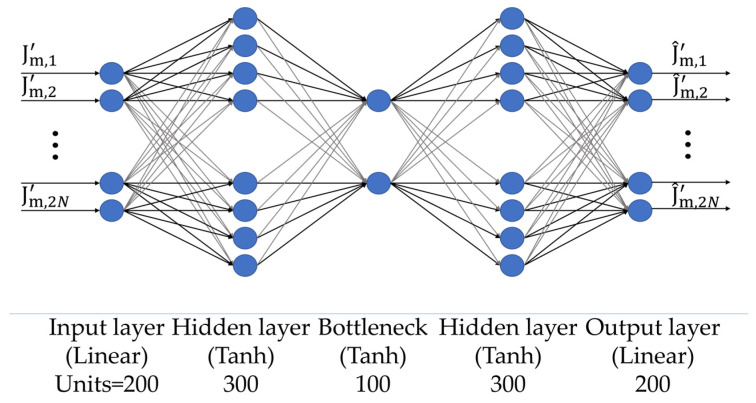
MLP architecture of the sAE.

The sAE architecture is represented in Figure 6. Like dAE, the network consists of 5 layers: 1 input layer, 3 hidden layers, and 1 output layer. The input layer is made of several nodes equal to the size of input data $J_{m}^{\prime }$, i.e., 2N = 200. The second layer has 300 neurons, the third layer has $b_{2}$ = 100 nodes (bottleneck), and the fourth layer has 300 nodes. Finally, the output layer has as many nodes as the input layer (2N = 200). A Tanh activation function is applied to the output of the second, third, and fourth layers, while a linear activation function is considered at the input and output layers.

**Figure 7:**
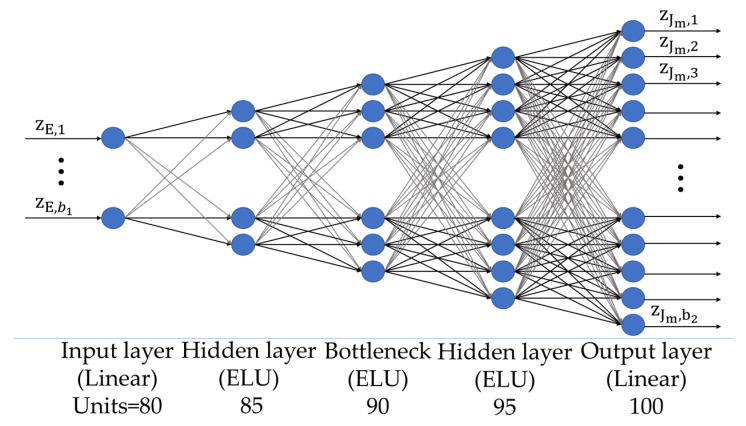
MLP architecture of the Σ network.

The structure of the Σ network displayed in Figure 7 comprises 9 layers: 1 input layer, 7 hidden layers, and 1 output layer. The number of nodes in the input layer is equal to the size of the latent representation of the data ($b_{1}$ = 12). The second to eighth layers are made of 30, 40, 50, 60, 70, 80, and 90 nodes, respectively, and the Exponential Linear Function Unit (ELU) activation function [50] with parameter α = 1 is exploited. Finally, the output layer has $b_{2}$ = 100 nodes and provides the latent representation of the source via a linear activation function.

The dAE, sAE, and Σ networks are trained separately. More specifically, dAE is provided with a dataset of $N_{tr}$ vector pairs ($E^{\prime },$ $E_{gt}^{\prime })$, where $E^{\prime }$ is the input noisy data vector and $E_{gt}^{\prime }$ is the desired noiseless output (ground truth data). Similarly, sAE is given, in the input, a dataset of $N_{tr}$ vector pairs ($J_{m}^{\prime },$ $J_{m,gt}^{\prime })$, where $J_{m}^{\prime }$ corresponds to the ground truth $J_{m,gt}^{\prime }$ by definition. Once the dAE and sAE networks are trained, their encoding paths are used to generate $N_{tr}$ vector pairs ($z_{E},$ $z_{J_{m}})$, which are the latent codes used for the training of the Σ network.

A mini-batch training strategy is exploited to balance efficiency and accuracy during the optimization process. In detail, the training dataset is split into smaller subsets (mini-batches), and, at every iteration, a new mini-batch is considered to calculate the model error and update the network coefficients. A complete pass through the whole training dataset is referred to as an epoch. At any iteration, the loss function minimized during the training is defined as the mean squared error (MSE) averaged over a mini batch with the size $N_{mb}$. By adopting a general notation where the prediction/ground truth pair is denoted by ($\hat{x}$, $x_{gt}$), the MSE is defined as

(13) $$\mathrm{MSE}=\frac{1}{N_{mb}}\overset{N_{mb}}{\underset{n=1}{\sum }}\frac{1}{Q}\|{\hat{x}}_{n}-x_{n,gt}{\|}^{2}$$

where ${\left\|\;\right\|}^{2}$ is the $\ell^{2}$-norm, and *Q* denotes the number of vectors ${\hat{x}}_{n}$ and $x_{n,gt}$ of the mini batch. The training of each network is carried out to reach sufficiently small values of the loss function. In this respect, a proper setting of the training options and hyperparameters is essential to achieve satisfactory performance. The settings found after parametric simulations and those that were considered in this study are summarized in Table 2. More specifically, the ADAptive Moment (ADAM) optimizer was selected and allowed to operate with an adaptive learning rate, starting from 10^−3^ and halving after a fixed number of epochs (see Table 2). Data shuffling every epoch was carried out up to the selected maximum number of epochs in order to avoid any bias that might arise from the order of the data. The L-SVD strategy in Figure 2 was implemented and tested in the Python language by using the Keras library [51,52] under the Google Colab environment. A Graphic Processing Unit (GPU) equipped with 15 GB of RAM was provided by the environment for the computations. The total training time was about 40 min for the dAE and sAE and about 1 h 10 min for the Σ network.

### 3.5. Performance Metric

The quality of the training process is appraised by analyzing the curves of the training and validation MSE (see Equation (13)) versus the number of epochs. As for the testing, the generalization capabilities of the L-SVD strategy are assessed in a quantitative way by evaluating the mean percentage error (MPE), which is a measure of the discrepancy between network predictions ${\hat{x}}_{n}$ and the desired outputs $x_{n,gt}$. The MPE is evaluated, in percentage, according to Equation (13), while replacing the ground-truth elements $x_{n,gt}$ used for the training with those used for the testing and $N_{mb}$ with $N_{test}$.

## 4. Numerical Results

This Section discusses the results of the numerical experiments firstly by dealing with the performance of the stand-alone dAE, sAE, and Σ networks and later on by considering the overall L-SVD strategy. Concerning the training and validation loss for the dAE network, very low training and validation-loss values (i.e., 4.42 × 10^−8^ and 4.47 × 10^−8^, respectively) are achieved after 5000 epochs, while no relevant overfitting is observed.

### 4.1. Performance of the dAE

**Figure 8:**
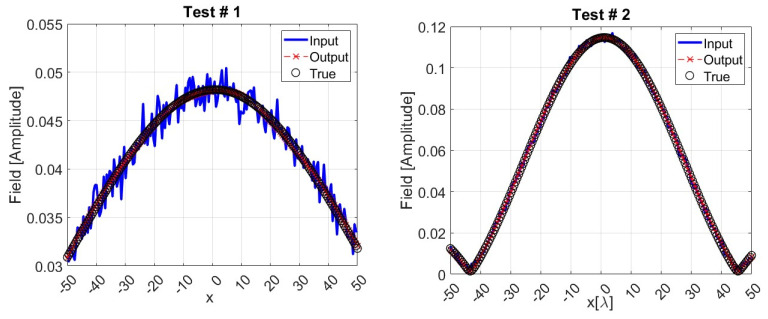
Two representative examples randomly selected in the test dataset, with SNR = 30 dB, showing the magnitude of inputs, outputs, and ground truth data for dAE.

The graphs illustrated in Figure 8 provide a representation of the dAE performance through two samples randomly chosen in the test dataset. In each graph, the amplitude of the noisy input field is compared to the network output and to the true (noiseless) field. As expected, the network output (red dashed line) is a denoised version of the input (solid blue line), and it reproduces the ground truth data (dotted line) in a satisfactory way.

In order to assess the dAE performance from a quantitative viewpoint, Table 3 summarizes the MPE values related to dAE inputs and outputs. The MPE of a TSVD-based denoising, achieved by projecting the input data on the first NDF singular vectors $u_{p}$ of the operator matrix $\underline{\underline{A}}$ (see Equation (9)), is included in the third column. Moreover, the TSVD-based MPE value obtained by considering the truncation index provided by the L-curve is reported in the fourth column. The numerical data suggest that the dAE allows for achieving a reduction in MPE (around 2.66) as compared to the input and also a better denoising performance than each TSVD solution. In this respect, it should be stressed that the L-curve criterion allows for achieving better TSVD performance, because the mean value of the optimal truncation index over the test dataset is equal to 9 and so is slightly larger than NDF.

### 4.2. Performance of the sAE

Regarding the training and validation loss for the sAE network, after 5000 epochs, they reach very small and similar values (i.e., 1.03 × 10^−7^ and 1.04 × 10^−7^, respectively), and only a negligible overfitting is observed.

**Figure 9:**
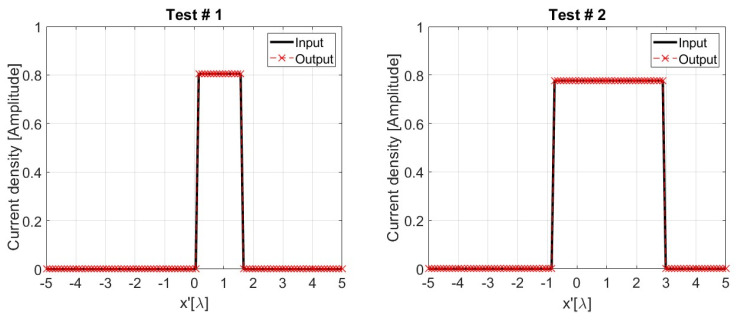
Two representative examples randomly selected in the test dataset, with SNR = 30 dB, showing the magnitude of the true source (input) and the one reconstructed via sAE (output). The current sources shown in the graphs are the ones generating the radiated fields in Figure 8.

Figure 9 shows that two source reconstructions are achieved via sAE. In this case, the input data are noiseless, and the current sources retrieved from their corresponding latent codes are in almost perfect agreement with the ground truth data. This claim is corroborated by the MPE evaluated over the test dataset, which is nearly equal to 0.1.

### 4.3. Performance of the Σ Network

After training the dAE and sAE, the data and source encoding paths are exploited to generate a dataset of latent codes, which are the input and output data required for the training and testing of the Σ network. In this regard, after 1000 epochs, acceptable values for the training and validation losses (i.e., 7.2 × 10^−5^ and 7.3 × 10^−5^, respectively) are achieved, and, as for the testing, an MPE value equal to 13.06 is obtained.

### 4.4. Performance of the Full L-SVD Network

**Figure 10:**
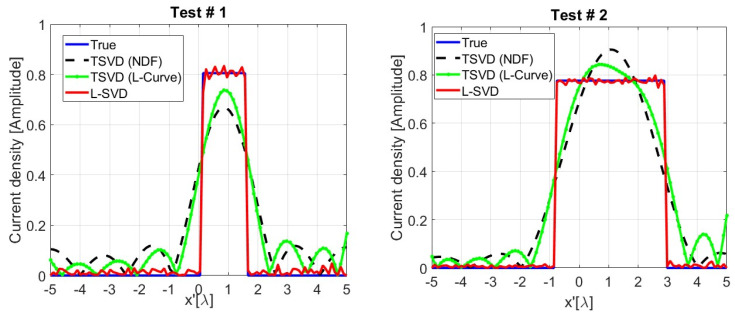
Two representative examples randomly selected in the test dataset, with SNR = 30 dB, showing the amplitude of the true source and those retrieved via TSVD and L-SVD. The true sources in the graphs are those previously shown in the examples of Figure 9.

The L-SVD inversion strategy is implemented by assembling the trained networks as per the green path in Figure 2. Therefore, the noisy data are encoded, converted to latent source codes via the Σ network, and finally decoded to retrieve the original sources. Two reconstruction tests showing the operation of the L-SVD and corresponding to data and sources formerly considered in Figure 8 and Figure 9 are reported in Figure 10. Here, the L-SVD source reconstructions are compared to both TSVD solutions as well as to the ground truth distributions. These results show that the sources retrieved via L-SVD better follow the true sources compared to the TSVD reconstructions. More specifically, the profiles retrieved via L-SVD are characterized by smaller oscillations, suggesting that it is possible to recover a larger number of high-frequency components of the unknown. The MPE values achieved by TSVD and L-SVD listed in Table 4 also confirm that the L-SVD considerably outperforms the TSVD inversion scheme. The oscillations occurring in the L-SVD reconstruction are related to the degree of approximation, offered by the NN, to the inverse link between data and unknowns.

### 4.5. Robustness of Noise in Data

A robustness analysis of the noise level in the radiated field data is now carried out to examine the possible limitations of the L-SVD strategy, which was previously trained on a dataset characterized by an SNR = 30 dB. In detail, additional test datasets, each made by 20,000 samples, are produced for progressively increasing noise levels (SNR = 30, 20, 10, and 0 dB). Then, the dAE and L-SVD source reconstruction strategies are tested for each SNR level, and the attained results are compared to the TSVD-based ones in Table 5 and Table 6, respectively. It is interesting to notice that, when the SNR departs from the value considered for the training (SNR = 30 dB), the denoising performance of dAE (see Table 5) and, consequently, the reconstruction capability of the L-SVD inversion (see Table 6) both degrade. In particular, higher MPEs are observed when the data are noisier (see SNR = 0 dB). This outcome confirms that the network generalization capabilities deteriorate in the case of very noisy measurements, if the network has been trained on cleaner data. Despite this, it turns out that the L-SVD strategy is more performing than TSVD, except for the case of very noisy data (SNR = 0 dB).

To check if the L-SVD performance can be made more robust to the noise in the radiated field, a new training dataset with 80,000 samples, here referred to as a mixed dataset, is built by accounting for different noise levels (SNR = 30, 20, and 10 dB). In detail, the dAE and Σ networks are re-trained by considering the mixed dataset with the same settings as in Table 1. Furthermore, since the sAE is appointed to reduce the dimensionality of the unknown space and is trained in the case of noiseless sources only, the sAE itself is not re-trained for this further testing. Table 7 and Table 8 summarize the MPE results achieved after testing the dAE and L-SVD strategies when trained on the mixed dataset. By comparing the data in these tables with their counterparts in Table 5 and Table 6, it can be established that accounting for more noise levels in the training phase slightly extends the generalization capabilities of the L-SVD, which now outperforms TSVD for every SNR level. It must also be noticed that L-SVD yields a slightly larger MPE at SNR = 30 dB (6.16 vs. 5.30 in Table 6), but such a minor performance worsening is compensated for by the improvement in reconstruction errors at the lowest SNRs.

**Figure 11:**
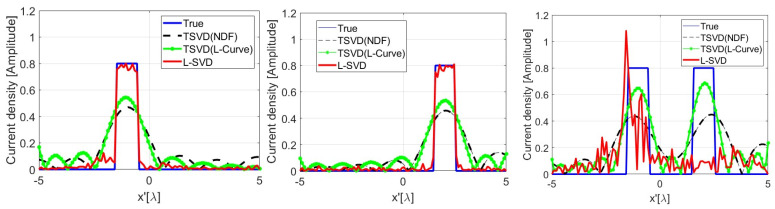
Illustrating the missing additivity property of linearity for L-SVD.

**Figure 12:**
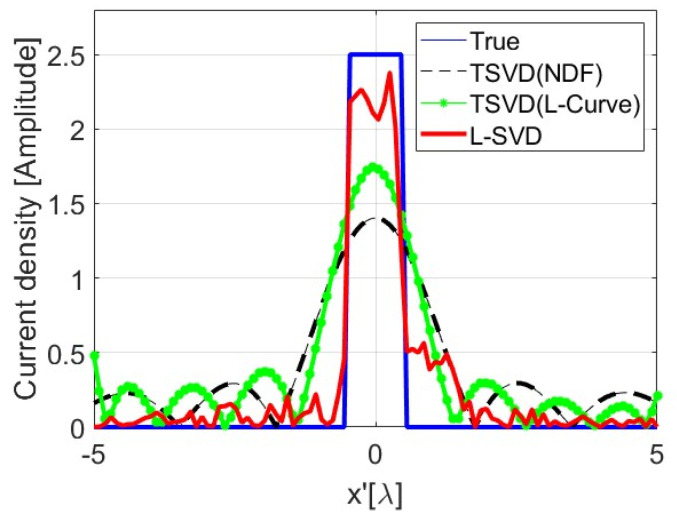
Illustrating the missing homogeneity property of linearity for L-SVD.

In order to show the loss of linearity when using a DNN as opposed to the TSVD case, in Figure 11, we display the reconstruction of two rectangular pulses whose amplitude and spatial extent are coherent with the training set. As can be seen, the reconstruction of each individual pulse is satisfactory, while, when the two pulses are simultaneously present in the scenario, the L-SVD fails: the additivity property of linearity is not met. Furthermore, in Figure 12, we depict the same reconstruction of one of the pulses of Figure 11 but with a different amplitude, which is not coherent with the exploited training dataset. As it can be seen, the L-SVD result is unsatisfactory, and the homogeneity property of linearity is again not met.

## 5. Discussion: Relevance of the Results and Potentials and Limitations of L-SVD

In the present paper, the L-SVD topology was applied, for the first time, to an electromagnetic inverse source problem, exploiting both amplitude and phase data with a radiated field collected in the far-field zone so as to reach a Fourier transform relation between data and unknowns. The results are of great interest, also for all those inverse problems governed by a Fourier–transform relationship, such as microwave imaging [53], computed tomography [54], magnetic resonance imaging [55], deconvolution [56], optics [57], geophysical imaging [58], and astronomy [59]. The results can be extended to cases where the amplitude and phase data are collected in the near-field zone [60], or where amplitude-only data are acquired in the far field [61]. The L-SVD approach can be also applied to solve inverse scattering problems under different measurement configurations not matching the far-field conditions [62,63].

Being based on the use of AEs, L-SVD has the potential to reach low-rank representations of data and unknowns. The advantage of such low-rank representations is that they are interpretable, that is, the physical meaning of the salient features of data and unknowns can be better highlighted, as pointed out above, since the idea underlying L-SVD is to offer a network topology close to the principles of classical SVD. Moreover, low-rank representations reduce the number of DNN parameters, especially as compared to fully connected networks, which has benefits in terms of faster training and inference times, a faster convergence of training due to fewer updates per epoch and possibly fewer epochs overall, a reduced risk of overfitting, more robust training, and implicit regularization offered by reduced dimensionalities. The reported results also highlight the noise-filtering capabilities of L-SVD, since, thanks to the reduction in data dimensionality, only the most significant features of the data are retained.

On the other hand, L-SVD might not be extensively beneficial across all of the above-mentioned types of problems, since, as already noted, the advantages of L-SVD are more pronounced in scenarios where capturing the low-rank structure of data is crucial. Nevertheless, there is limited research, and few benchmarks are available for L-SVD compared to more conventional neural network architectures, so that a full understanding of the L-SVD performance is not possible for the time being.

## 6. Conclusions

This work analyzed the application of the L-SVD strategy for solving the electromagnetic inverse source problem. L-SVD is a generalization of SVD to the case of the non-linear modelling of the inverse problem that builds the solution in three basic steps: (i) a representation of the radiated field into a data-latent code; (ii) a conversion of the data-latent code into a source-latent code; (iii) a decoding of the source-latent code. Here, L-SVD is used to solve a linear inverse problem.

Thanks to its capability of accommodating a priori information on the set of relevant sources to be reconstructed (not just simply belonging to a subspace) and on the noise level in the data, different from TSVD, it was shown that, for a dataset relevant to a 2D scalar geometry and far-zone conditions, L-SVD provides better results than classical TSVD, even if its performance exhibits a stronger dependence on the noise in the data. Despite this, L-SVD yields lower reconstruction errors if compared to TSVD, and it allows for retrieving the higher frequency spectrum components of the source. The reason for the better performance of L-SVD stems from the fact that, different from TSVD, it conducts an intrinsic non-linear processing of the data. However, it must be stressed that L-SVD is a data-driven reconstruction approach, and thus it does not work properly when no a priori information about the problem is available. This entails that L-SVD works well only with the class of sources exploited in the training stage. The major improvements of this approach are thus the capability of generalizing the TSVD approach to a non-linear problem and of exploiting the a priori information on the source to improve the results against the standard TSVD, giving, at the same time, a meaningful interpretation of the network layout.

In this paper, specific training of the L-SVD network for the reconstruction of a solitary source was considered. Facing the reconstruction of multiple sources is, however, also possible, provided that more a priori information and possibly a different NN topology can be exploited.

Future research activity will be focused on the application of the L-SVD approach to the electromagnetic inverse scattering problem. In particular, future analyses could be worked out for inverse problems, whose classical subspace formulation involves non-linear operators, extending the work here.

## Figures and Tables

**Table 1:** Simulation parameters for the inverse source problem.

Parameter	Value
Source semi-extension	$a^{\prime }=5\;\lambda$
Observation domain semi-extension	a=50 λ
Number of source points	N=100
Number of measurement points	M=200
Distance between domains	z=160 λ

**Table 2:** Training options and hyperparameters’ settings for dAE, sAE, and Σ network.

Option/Parameter	dAE	sAE	Σ
Optimizer	ADAM	ADAM	ADAM
Initial learning rate	10^−3^	10^−3^	10^−3^
Learning-rate drop period	500	500	250
Learning-rate drop factor	0.5	0.5	0.5
Mini-batch size	640	640	64
Max. number of epochs	5000	5000	1000

**Table 3:** MPE values for dAE input/output and TSVD when SNR = 30 dB.

Input	Output	TSVD (NDF)	TSVD (L-Curve)
3.16	0.50	6.85	0.71

**Table 4:** MPE values for TSVD and L-SVD inversion (SNR = 30 dB).

TSVD (NDF)	TSVD (L-Curve)	L-SVD
33.15	30.43	5.30

**Table 5:** MPE values for dAE input/output and TSVD for different noise levels—training at SNR = 30 dB.

SNR [dB]	Input	Output	TSVD (NDF)	TSVD (L-Curve)
30	3.16	0.50	6.85	0.71
20	10.00	1.54	7.04	2.18
10	31.65	4.85	8.76	6.50
0	100.04	15.27	18.62	18.94

**Table 6:** MPE values for TSVD and L-SVD inversion at different noise levels—training at SNR = 30 dB.

SNR [dB]	TSVD (NDF)	TSVD (L-Curve)	L-SVD
30	35.15	30.43	5.30
20	35.19	31.90	7.56
10	35.54	33.96	17.68
0	38.94	39.38	46.73

**Table 7:** MPE values for dAE input/output and TSVD for different noise levels—training on the mixed dataset.

SNR [dB]	Input	Output	TSVD (NDF)	TSVD (L-Curve)
30	3.16	0.84	6.85	0.71
20	10.00	1.37	7.04	2.18
10	31.65	3.73	8.76	6.50
0	100.04	11.54	18.62	18.94

**Table 8:** MPE values for TSVD and L-SVD inversion for different noise levels—training on the mixed dataset.

SNR [dB]	TSVD (NDF)	TSVD (L-Curve)	L-SVD
30	35.15	30.43	6.16
20	35.19	31.90	7.54
10	35.54	33.96	14.73
0	38.94	39.38	31.08

## Data Availability

Data are unavailable due to privacy restrictions.
